# Alternation of apoptotic and implanting genes expression of mouse embryos after re-vitrification

**Published:** 2016-08

**Authors:** Nasrin Majidi Gharenaz, Mansoureh Movahedin, Zohreh Mazaheri, Shahram Pour beiranvand

**Affiliations:** *Department of Anatomical Sciences, Faculty of Medical Sciences, Tarbiat Modares University, Tehran, Iran.*

**Keywords:** *Vitrification*, *Embryo*, *Apoptosis*, *Gene*

## Abstract

**Background:**

Nowadays, oocytes and embryos vitrification has become a routine technique. Based on clinical judgment, re-vitrification maybe required. But little is known about re-vitrification impact on genes expression.

**Objective:**

The impact of re-vitrification on apoptotic and implanting genes**,*** Bax, Bcl-2* and *ErbB4*, at compaction stage embryos were evaluated in this study.

**Materials and Methods:**

In this experimental study, 8 cell embryos (n=240) were collected from female mature mice, 60-62 hr post HCG injection. The embryos were divided randomly to 3 groups included: fresh (n=80), vitrified at 8 cell stage (n=80), vitrified at 8 cell stage thawed and re-vitrified at compaction stage (n=80). Embryos were vitrified by using cryolock, (open system) described by Kuwayama. Q-PCR was used to examine the expression of *Bax, Bcl2 ErbB4* genes in derived blastocysts.

**Results:**

Our result showed that expanded blastocyst rate was similar between vitrified and re-vitrified groups, while re-vitrified embryos showed significant decrease in expanded blastocyst rate comparing with fresh embryos (p=0.03). In addition, significant difference was observed on apoptotic gene expression when comparing re-vitrified and fresh embryos (p=0.004), however expression of *Bax *and* Bcl-2* (apoptotic) genes didn't demonstrate a significant difference between re-vitrified and vitrified groups. The expression rate of *ErbB4*, an implantation gene was decreased in re-vitrified embryos comparing with fresh embryos (p=0.003), but it was similar between re-vitrified and vitrified embryos.

**Conclusion:**

Re-vitrification can alter the expression of *Bax, Bcl-2* and *ErbB4* genes and developmental rate of mouse embryos in compaction stage.

## Introduction

Oocyte and embryo cryopreservation can contribute to a reduction of ovarian hyper stimulation syndrome (OHSS), improvement of cumulative pregnancy rates and reduction of multiple gestations during assisted reproductive techniques (1-4). Furthermore, several findings also confirm a better obstetrical outcome of children being born after a frozen embryo transfer (FET) (5-7). Additionally, this method is useful to fertility preservation of patients who are candidate for chemotherapy or radiotherapy (8). 

Occasionally, unexpected extra embryos are available after thawing the embryos and re-cryopreservation after frozen-thawed embryos transfer is inevitable (9). These embryos could be re-vitrified for a future transfer. It has been revealed that previously frozen embryos during in vitro fertilization (IVF) could be thawed for genetic evaluation and re-vitrified for transfer at a later date (10). Providing the option to re-cryopreserve a patient’s previously stored embryos, may encourage more patients to take a cautious approach with regard to number of embryos transferred. Farhat *et al* reported first successful clinical pregnancy following transfer of re-vitrified embryos (11). 

Ito *et al* evaluated impact of re-vitrification embryos (2 cell stage, 4 cell stage, and morula, blastocyst stage) on the same stage on the embryos developmental competence. They reported re-vitrification with cryotop did not affect the developmental ability of mouse embryos (12). Shehan also reported similar results. But they did not evaluate the re-vitrification effect on gene expression. Severe injuries may occur at all phases of these procedures (13). Hence, cryopreservation may cause fragmentation in frozen thawed embryos (14-16). Moreover, profile of apoptotic and implanting genes can be affected by vitrification procedure (17, 18).

Several publications postulated that vitrification could induce apoptosis in vitrified embryos and alter the expression of apoptosis related genes (17-20). Members of* Bcl-2* gene family are known to have an important role in regulating apoptosis. *Bcl-2* is known for its anti-apoptotic activity and responsibility in cell survival, whereas *Bax* is pro-apoptotic and responsible for the induction of cell death (20). *ErbB4* is a gene involved in implantation which interacts with heparin binding-epithelial growth factor (HB-EGF) to mediate attachment of the blastocyst to uterine luminal epithelium (21, 22). There is insufﬁcient data on the effect of repeated use of vitriﬁcation and slow freezing on genes expression; there have been several studies on the efﬁciencies of re-vitriﬁcation and double slow freezing on embryo viability (11, 23,25). 

Therefore re-vitrification effects on developmental rate and *Bax, Bcl-2* and *ErbB4* genes expression pattern were investigated.

## Materials and methods

This experimental study was performed on mouse embryos. Female NMRI mice (6-8 wks) were purchased from the Pasteur Institute (Tehran, Iran). The mice were housed in a room under standard laboratory conditions (12 hr light/dark at 22^o^C) with free access to water and standard food. The current study was conducted under the protocol approved by the animal experimentation committee of Medical Sciences Faculty in Tarbiat Modares University. 

**Embryo collection and culture**

Mice were super ovulated by an intra-peritoneal (IP) injection of 7.5 IU pregnant mare’s serum gonadotropin (PMSG; Intervet Inc, Netherlands) followed 48 hr later by 7.5 IU human chorionic gonadotropin (HCG; Pregnil; Organon, Oss, Netherlands). The mice were mated with a single male of the same age (6-8 wks old). Presence of vaginal plugs was a confirmation of a pregnancy. The pregnant female mice sacrificed by cervical dislocation 60-62 hr post HCG injection to collect 8-cell embryos (26). 

240 embryos were divided to three groups. Group 1; 8-cell embryos were cultured in HTF (Geneocellideal, Iran) medium supplemented with 10% human serum albumin (HSA, Biotest, Germany) until 72 hr. Group 2; 8-cell embryos were vitrified- warmed at 8-cell stage, then were cultured until 72 hr. Group 3; initially 8-cell embryos were vitrified- warmed. After 6-8 hr, alive embryos re-vitrified at compaction stages. Then, compacted embryos were cultured until 64 hr.

**Vitrification procedure**

Mouse embryos were vitrified by a two-step procedure with vitrification kit (Geneocellideal, Iran) using the cryolock (Biotech, USA) as a carrier, as described by Kuwayama (27). Embryos were initially equilibrated in equilibration solution (ES) at room temperature for 5-15 min. ES included 7.5% (v/v) ethylene glycol (EG, Sigma Germany) and 7.5% (v/v) dimethylsulfoxide (DMSO, Sigma Germany) dissolved in HTF medium supplemented with 10% HSA. 

After initial shrinkage, the embryos regained their original volume and were transferred to vitrification solution (VS) for 1 min. Vitrification solution consisting of 15% (v/v) EG, 15% (v/v) DMSO and 0.5 mol/L sucrose (Sigma Germany) dissolved in HTF medium supplemented with 10% HAS. Within less than 60 sec, 2-3 embryos in minimal VS (<1.0 µl) were placed on to the inner surface of the cryolock carrier. The cryolock was plunged vertically into liquid nitrogen. 

**Warming technique**

After cryo-storage, the embryos were warmed using a three steps dilution procedure with vitrification kit (Geneocellideal, Iran). Briefly, the cryolock containing the embryos were removed from liquid nitrogen and dipped into T1 solution (Geneocellideal, Iran) containing 1.0 mol/L sucrose at 37^o^C. After 1 min equilibration in T1 solution, the embryos were moved into T2 solution containing 0.5 M sucrose for 3 min. Then the embryos were moved into T3 solution containing 0.25 M sucrose for 3 min. Finally the embryos were washed by HTF medium. Re-vitrification procedure was done by a two-step procedure as described by Kuwayama.

**Assessment of embryo survival**

Viability of warmed embryos was determined based on visual examination of the integrity of embryo membrane, zona pellucida and the normality of the cytoplasm two hours after warming. During in vitro culture, embryo development was evaluated every day. 

**Molecular assessment by quantitative polymerase chain reaction (qPCR)**

Total RNA was isolated from the embryos in each group using QIAzol (Qiagen, Germany) according to the manufacturer’s recommendations. To eliminate any genomic DNA contamination, RNA samples were treated with DNase using a kit (EN0521; Fermentas). The RNA concentration was determined by spectrophotometery, and the RNA samples were stored at -80^o^C until use. The cDNA was synthesized in a total volume of 20 µl containing 5 µg total RNA either with reverse transcriptase (+RT cDNA) or without the enzyme (-RTcontrol) using the cDNA kit (Fermentas, EU) and stored at -20^o^C until use (26).

All experiments were carried out in triplicate. For PCR reactions, primers were designed by the NCBI website and gene runner software and synthesized by Cinnagen (Iran) (Table I). PCRs were performed using Master Mix and SYBR Green in an Applied Biosystems, StepOne™ thermal cycler (Applied Biosystems, USA). The PCR program started with an initial melting cycle for 5 minutes at 95^o^C to activate the polymerase, followed by 40 cycles of melting (15 sec at 95^o^C), annealing (30 sec at 58^o^C) and extension (15 sec at 72^o^C). The quality of the PCR reactions was confirmed by melting curve analyses. 

Efficiency was determined for each gene using a standard curve (logarithmic dilution series). For each sample, Glyceraldehyde 3-phosphate dehydrogenase (GAPDH) was used as the reference gene and target gene were amplified in the same run. Reference gene was approximately equal. The target genes were normalized to a reference gene and expressed relative to a calibrator (Group 1 as control group).

**Statistical analysis**

Developmental rates of vitrified-warmed embryos were analysed with the Chi square test. qPCR data were presented as mean±SD and were analysed using One-way analysis of variance (ANOVA) followed by Tukey’s post hoc test. p<0.05 were considered statistically significant.

## Results

**Survival rate finding in experimental groups**

There was significant difference between re-vitrified embryos (87.5%) and fresh embryos (100%) regarding survival rate (p=0.03), but survival rates of vitrified and re-vitrified embryos didn’t show any significant difference (Table II). 

**Development to expanded blastocyst**

Based on the current findings, expanded blastocyst rate didn't show any significant difference between vitrified and re-vitrified groups. However, there was significant difference between re-vitrified embryos (82.9%) and fresh embryos (92.5%) (p=0.03) (Table II).

**Degeneration rates investigation**

The results of degeneration rate showed no significant differences between vitrified and re-vitrified groups. While, there was significant difference between re-vitrified and fresh embryos (p=0.03) (Table II).

**Profile of genes expression**

**Figure 1:**
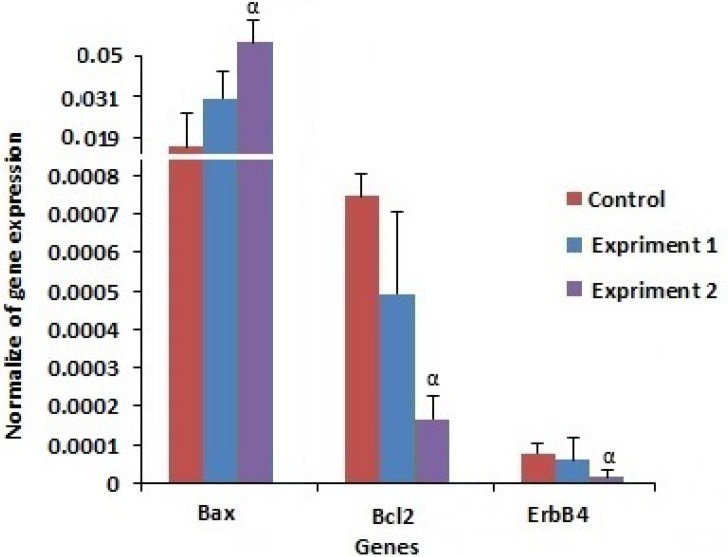
Profile of apoptotic and implanting genes expression. l; fresh embryos, 2; vitrified 8 cell embryos , 3; vitrified 8 cell embryos and re-vitrified at compact stage. α indicated valued differs with control (p<0.05

Expression of *Bax* (as pro apoptotic gene) was significantly higher in re-vitrified embryos compare to fresh embryos (p=0.004). Moreover,* Bcl-2* (as anti-apoptotic gene) expression rate, was lower in re-vitrified embryos compare to fresh embryos (p=0.004). Meanwhile, expression rate of *Bax* and* Bcl-2* was similar between re-vitrified and vitrified groups. The expression rate of *ErbB4*, an implantation gene, was similar between re-vitrified and vitrified groups. Although it was significantly lower (p=0.003) in re-vitrified embryos compared to fresh embryos (Figure 1).

**Table I:** Genes, primers, and sizes of amplification products (bp) for quantification of gene expression by real-time quantitative polymerase chain reaction

**Gene**	**Primer sequence**	**Length (bp)**	**Code number**	**Tm (** ^o^ **C)**
*GAPDH*	F:5´-GACTTCAACAGCAACTCCCAC-3´ R:5´- TCCACCACCCTGTTGCTGTA-3´	125	NM_001289726.1	80
*Bax*	F:5´- CGGCGAATTGGAGATGAACTG-3´ R:5´-GCAAAGTAGAAGAGGGCAA-3´	161	XM_006540584.1	83.5
*Bcl2*	F:5´- ACCGTCGTGACTTCGCAGAG -3´ R:5´- GGTGTGCAGATGCCGGTTCA -3´	239	NM_009741.1	84
*ErbB4*	F:5´-TACGAGCCTGCCCAAGTTC- 3′ R:5´- GTGCCGATTCCATCACATCCT- 3′	103	XM_006536907.1	76.5

**Table II:** Embryonic developmental rate in 3 groups after 72 hr culture

**Groups **	**Survival rate (%)**	**Blastocyst formation (%)**	**Degeneration (%)**
Control	80 (100)	74 (92.5)	6 (67.5)
Experiment 1	71 (88.8^a^)	71 (88.8)	9 (11.2)
Experiment 2	70 (87.5^a^)	70 (82.9^ a^)	10 (17.1^ a^)

1; fresh embryos, 2; vitrified 8 cell embryos, 3; vitrified 8 cell and re-vitrified at compact

α on error bar indicated valued differs with group 1 (p<0.05).

Developmental rates of embryos were analysed with the Chi square.

**Figure 2:**
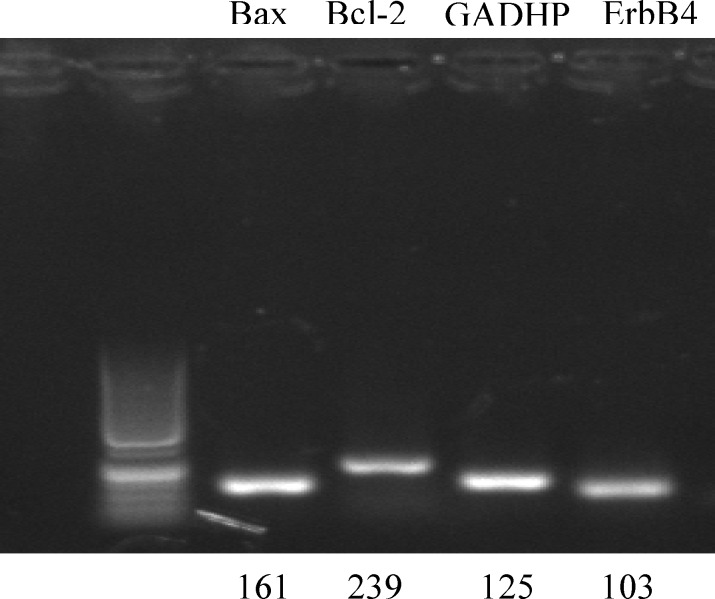
Expression of *Bax*, *Bcl*-2, *ErbB4*, and *GAPDH* genes

## Discussion

This research evaluated the impact of re-vitrification on developmental rate and profile of apoptotic and implanting genes expression in mouse embryos. Result of the current study demonstrated clearly that in vitro development in re-vitrified embryos reduced comparing to fresh embryos. However some investigator reported that re-vitrification did not negatively affect the developmental rate of embryos (12, 25, 28). 

The reasons for this difference might be vitrification device, concentration of cryoprotectant, stage of embryos, species, and culturing time between vitrification and re-vitrification. Severed osmotic stress during vitrification and warming may alter integrity of embryo morphology and cellular actin filament organization (19). The factors which have been known to affect the cryopreservation of embryos and subsequently their developmental rate are; types of cryoprotectants, cooling and warming rates, dilution methods, embryo stage and quality of embryos (29). 

Some investigators demonstrated that supplementing the cryopreservation media with an antioxidant reduces oxidative stress and increases the survival rates of embryos (30). Shehan *et al* added ascorbate to cryopreservation solution as an antioxidant, then re-vitrified mouse embryos at pronuclear stage, 2-cell stage, 8-cell stage and blastocyst stage and reported that re-vitrification of embryos didn't affected the survival rates and degeneration rates of embryos (25). An antioxidant treatment can be useful for development of embryos and cells differentiation (30). It is hypothesized that antioxidant can scavenge free radicals, which could damage DNA and the phospholipid membrane of the embryo (31). 

Presence of an antioxidant in cryopreservation solution maybe explanation for the disparity between our results and Shehan's data. Gayar *et al* reported that in vitro and in vivo developmental rate of mouse blastocysts vitrified once or twice, did not show any significant differences with fresh embryos. Additionally, the viability of the embryos after single or repeated vitrification, was virtually identical. A third time vitrification, however, was not tolerated by the majority of embryos (32). This may be indicative of embryos experiencing some stress during successive vitrification procedures. It was reported that repeated freezing mouse embryos without a culture time intervention between each cycle after thawing contained lower mean cell numbers at hatching compared to unfrozen embryos (27). 

It seems that repeated exposure of embryos to high cryoprotectant concentration could have a toxic effect on development of embryos. High concentration of cryoprotectants could alter enzymatic activity and plasma membrane integrity of embryos (33-35). Additionally, disturbance in sodium-potassium pump during blastocyst formation may be a cause of cellularity and developmental rates reduction after vitriﬁcation and re-vitriﬁcation (36). Also, the exposure of blastocysts to cryoprotectant decreases the survival rates and increases the DNA-fragmented nuclei in embryos (37). Fundamental mechanisms of oocytes degeneration and embryo fragmentation is apoptosis (20). 

Apoptosis is a regulated program that initiate cell death. Memebers of *Bcl-2* genes family play an important role in apoptosis regulation. Hence, we evaluated the possible impact of re-vitrification on expression of *Bax *and *Bcl-2*. The current findings demonstrate clearly that re vitrification down regulated *Bax* and up regulated *Bcl-2. Bcl-2* is anti-apoptotic and is responsible for cell survival, whereas *Bax* is pro-apoptotic and induced cell death (20). 

Therefore, the decreased developmental capability of re-vitrified embryos, has a relation to regulation of apoptosis pathways. Similar to the current study, Dhali *el al* reported that lower survival and development of vitrified zygotes and 2-cell embryos may be attributed to the occurrence and regulation of embryonic apoptosis. The results of Dalhi's studied, clearly indicated a strong relationship between the compromised developmental competence and altered transcriptional activities of *Bax*, *Bcl-2*, and *p53* genes in the treated embryos (17). 

We also evaluated the possible impact of re-vitrification on implanting related gene *ErbB4*. It was demonstrated that expression of *ErbB4* remained similar in re-vitrified embryos compared to vitrified embryos, however, re-vitrification down regulated *ErbB4* gene expression. Interaction between ErbB4 and HB-EGF mediated the initial attachment of blastocyst to uterine luminal epithelium (22, 38). Signalling by HB-EGF back to the embryo, in turn, activates the program of trophoblast differentiation required for adhesive functions during subsequent attachment and invasion (22). 

Successful implantation is an absolute necessity for the reproduction of species. The process by which a foreign blastocyst is accepted by the maternal endometrium is complex and requires interplay of many systems. Many genes, of embryonic and endometrial origin are involved in this process (38-40). Therefore, changes in the gene expression being involved in this process should be further evaluated. Successful live birth from re-vitrified pronucleus and blastocyst have been reported after re-slow freezing embryos; therefore, progress has already been made in this aspect of clinical treatment (11, 23).

Consequently, the re-freezing of blastocysts has resulted in a successful birth (23). In conclusion the current findings clearly demonstrate that re-vitrified and vitrified embryos are similar in developmental rate and expression of *Bax, Bcl2* and *ErbB4* genes. However, blastocyst rate and the expression of* Bax, Bcl2* and *ErbB4* genes differ significantly between re-vitrified and fresh embryos. The impacts of the current findings should be further evaluated on obstetrical outcome and long term follow up of the children being born after re-vitrification.

## Conclusion

Re-vitrification can alter the expression of *Bax, Bcl-2* and *ErbB4* genes and developmental rate of mouse embryos in compaction stage.

## note

***This article extracted from M.Sc. thesis. (Nasrin Majidi Gharenaz)***
